# Prevalence of antibodies to human immunodeficiency virus, hepatitis B, and hepatitis C in prisoners in Libya

**DOI:** 10.3402/ljm.v7i0.19713

**Published:** 2012-12-18

**Authors:** Hisham Ziglam, Abdel-Aziz Zorgani, Ahmed Balouz, Abdel Hafidh Abudher, Omar Elahmer

**Affiliations:** Department of Infectious Diseases, Central Hospital Tripoli, Libya; Department of Microbiology and Immunology, Faculty of Medicine, Tripoli University, Tripoli, Libya; Department of Chest Medicine, Aboseta Hospital Tripoli, Libya; National Centre of Disease Control, Tripoli, Libya

Prison inmates are reported to exhibit higher rates of disease morbidity, mortality, and health care utilization than the general population (1, 2). The rates of infectious diseases such as hepatitis B virus (HBV), hepatitis C virus (HCV), and human immunodeficiency virus (HIV) are reported to be particularly elevated in prison (3–5). This excess is largely attributed to the high concentration of inmates with a history of injection drug use (6, 7). Other risk factors that may place inmates at increased risk of HCV either prior to or following incarceration include intranasal cocaine use, prostitution, and other high-risk sexual activity (6, 7). The situation regarding blood-borne viruses and intravenous drug users (IDUs) in prisons in low- and middle-income countries is unclear because accurate data are limited and difficult to access. Indications that the situation might be more detrimental than in high-income countries include the fact that 90% of HIV cases live in low-income countries (8), that HIV prevalence is often higher in the general community in low-income countries than in high-income countries (8), and that three-quarters of the estimated 13 million IDUs live in low- and middle-income countries (9). Because of the seriousness of HBV, HBC, and HIV infections among inmates, it is important to know the prevalence of these infections.

We determined the frequency of HIV, HBV, and HCV in 6371 male prisoners aged 16 and above examined between January and December 2006 in five prison blocks in the western part of Libya (Table 1). The study protocol was approved by the Centre of Disease Control Research Committee Board. The hepatitis B surface antigen (HBsAg) (ELISA, AXSYM, Abbott, Chicago, IL, USA), the HCV antibodies (ELISA, AXYSM version 3.0, Abbott, Chicago, IL,USA), and the HIV antibody tests were conducted at the reference laboratory of the Centre of Disease Control; positive HIV cases were confirmed by western blot (Genlab Diagnostic, Redwood City, CA, USA). The frequencies were 6.9% for HBsAg, 23.7% for the hepatitis C virus, and 18.2% for HIV. Nine hundred seventy-seven prisoners (15.3%) had positive results for more than one of the infections, and 95 (1.5%) had positive results for three viruses. Eighty-four percent of HIV-positive prisoners were hepatitis C–positive as well (Table 2).

**Table 1:** Socio-demographic background among male prisoners

	Nationality^b^			Marital status^d^		
				
Age^a^	Libyan (%)	Non-Libyan (%)^c^	*P*	Married (%)	Not married (%)	*P*
16–25	453 (7.4)	911 (14.8)	<0.05	194 (3.2)	1170 (19.1)	<0.05
26–40	2514 (40.9)	1474 (24)	<0.05	1156 (18.9)	2832 (46.1)	<0.05
>40	484 (7.9)	303 (5)	<0.05	643 (10.5)	144 (2.3)	<0.05
Total	3451 (56.2)	2688(43.8)		1993 (32.5)	4146 (67.5)	

a 233 prisoners with unidentified age.

b 215 prisoners with unknown nationality.

c 104 prisoners with unknown marital status.

d 96.7% of non-Libyan prisoners are of African origin.

**Table 2:** Seroprevalence of antibodies to human immunodeficiency virus (anti-HIV), hepatitis B surface antigen (HBsAg), and antibodies to hepatitis C virus (anti-HCV) in prisoners

Age group (years)	HBsAg positive		*P*	Anti-HCV		*P*	Anti-HIV		*P*
		
	Libyan^a^ (%)	Non-Libyan (%)		Libyan (%)	Non-Libyan (%)		Libyan (%)	Non-Libyan (%)	
16–25	21 (0.3)	44 (0.7)	<0.05	66 (1.1)	18 (0.3)	<0.05	48 (0.8)	26 (0.4)	<0.05
26–40	154 (2.5)	122 (2)	<0.05	1060 (17.3)	78 (1.3)	<0.05	789 (12.9)	104 (1.7)	<0.05
>40	32 (0.6)	38 (0.6)	<0.05	180 (2.9)	59 (0.9)	<0.05	132 (2.2)	29 (0.5)	<0.05

a 96.7% of non-Libyan prisoners are of African origin.

These results showed a significantly higher seroprevalence of HIV and HCV among the prison inmates in Libya as compared with the seroprevalence of these infections previously reported in the general healthy group in Libya (10, 11) or in prisons in developed countries (12).

Many studies from different parts of the world indicated that the prisoners represent a high-risk group for blood-borne diseases. Homosexuality and high-risk behaviors among prisoners may contribute to the transmission of these diseases. However our study neither allows us to determine whether the inmates acquired the diseases while in prison nor does it provide direct evidence of transmission of infectious diseases in prison. Furthermore, as a rule, there are inadequate medical facilities and staff in the prisons in Libya, and access to appropriate care outside the Libyan prison system is very difficult for the inmates. This epidemiological study represents a disturbing reality and is a public health issue. It is clear that inmates have a substantial risk of contracting these infections while they are in prisons. The current study underscores a critical need for local prevention activities. The urgency is augmented by the marked rise in HIV sero-incidence documented among IDUs in Libya and is surely a harbinger of worsening conditions for a variety of opportunistic infections and other sexually transmitted diseases.

*Hisham Ziglam* Department of Infectious Diseases, Central Hospital Tripoli, Libya Email: hisham.ziglam@gmail.com*Abdel-Aziz Zorgani* Department of Microbiology and Immunology, Faculty of Medicine, Tripoli University, Tripoli, Libya*Ahmed Balouz* Department of Chest Medicine, Aboseta Hospital Tripoli, Libya*Abdel Hafidh Abudher and Omar Elahmer* National Centre of Disease Control, Tripoli, Libya
